# Incorporating Squat-Based Training into the Warm-Up Twice Weekly Improves Sprint, Jump, and Change-of-Direction Performance in Young Soccer Players

**DOI:** 10.3390/sports14010040

**Published:** 2026-01-14

**Authors:** Okba Selmi, Hamza Marzouki, Mohamed Amine Rahmoune, Elena Adelina Panaet, Bogdan Alexandru Antohe, Cristina Ioana Alexe, Ana Maria Vulpe, Anissa Bouassida

**Affiliations:** 1 Department of Human and Social Sciences, High Institute of Sport and Physical Education of Kef, University of Jendouba, Kef 7100, Tunisia; okbaselmii@yahoo.fr (O.S.); hamzic_30@hotmail.com (H.M.); amine.iyad2040@gmail.com (M.A.R.); bouassida_anissa@yahoo.fr (A.B.); 2 High Institute of Sport and Physical Education of Sfax, University of Sfax, Sfax 3000, Tunisia; 3 Department of Physical and Occupational Therapy, “Vasile Alecsandri” University of Bacău, 600115 Bacău, Romania; panaet.adelina@ub.ro (E.A.P.); antohe.bogdan@ub.ro (B.A.A.); 4 Department of Physical Education and Sports Performance, “Vasile Alecsandri” University of Bacău, 600115 Bacău, Romania; zaharia.ana@ub.ro

**Keywords:** team sports, athletic conditioning, explosive strength, neuromuscular readiness, internal load monitoring

## Abstract

Understanding the long-term effectiveness of warm-up strategies is essential for enhancing neuromuscular performance in youth soccer players. This study examined the long-term effects of integrating squat exercises into the final phase of the warm-up over nine weeks on sprint, jump, change-of-direction (COD), and aerobic performance in youth soccer players. Twenty-four male U17 players were randomly assigned to either a squat-based warm-up (experimental group [EG]) or a rondo-based warm-up (control group [CG]). The EG trained twice weekly using 3–4 sets of 4–12 repetitions at progressively increasing intensities (50–85% of 1-RM). Performance was assessed pre- and post-intervention using 10 and 30 m sprint, squat jump (SJ), countermovement (CMJ), standing long jump (SLJ), 5-jump (5JT), T-half (COD), and VAMEVAL tests. The EG showed small to large significant gains in sprint (10 m: −2.21%, Cohen’s d [d] = 1; 30 m: −1.6%, d = 0.58), jumping (SJ: +9.29%, d = 1.23; CMJ: +12.08%, d = 1.83; SLJ: +7.14%, d = 0.8; 5JT: +2.33%, d = 0.32), and COD (−1.41%, d = 0.32), while aerobic endurance showed no significant change (*p* > 0.05). The CG showed no significant improvements (*p* > 0.05). Overall, integrating brief, progressive squat exercises at the end of warm-ups twice weekly led to chronic improvements in explosive neuromuscular performance, with minimal impact on aerobic endurance.

## 1. Introduction

Modern soccer demands that players perform repeated explosive actions, such as sprints, jumps, changes of direction (COD), and physical duels [1]. These qualities are essential for soccer performance, particularly during decisive phases of a match, where speed and power often determine the outcome [2]. In this context, the warm-up represents an essential component of athletic preparation, aimed at reducing injury risk while enhancing subsequent physical performance [3,4,5].

Traditionally, warm-ups include a general phase (light jogging, joint mobility) and a specific phase involving ball exercises [6]. Many coaches prefer to include a rondo in the final stage of the warm-up, as it stimulates reactivity, coordination, and passing speed under pressure [7,8,9]. This drill also enhances decision-making and perception-action coupling, providing a dynamic and engaging activity closely resembling match demands [7]. These well-established benefits make the rondo an important component of soccer training, particularly for developing technical and tactical skills in youth players [7]. Rondo drills may also promote teamwork, communication, and rapid problem-solving under time pressure.

Several alternative warm-up strategies have been proposed that incorporate strength exercises during the final phase of the warm-up [3]. When performed consistently over time, such exercises may act as repeated low-volume, high-intensity stimuli capable of inducing chronic neuromuscular adaptations, progressively enhancing force production, motor unit recruitment, and intermuscular coordination, factors critical for explosive actions in soccer [3,10,11]. Recent evidence in male adolescent soccer players supports the feasibility and effectiveness of frequent, time-efficient neuromuscular micro-doses in-season, showing that weekly high-frequency, strength-based training (e.g., plyometrics) can enhance physical performance outcomes while helping to manage muscle soreness [12]. It is important to distinguish these training-induced adaptations from acute post-activation potentiation (PAP) or post-activation performance enhancement (PAPE) effects, which reflect transient responses following a single conditioning stimulus rather than long-term adaptations [5,6,13].

Despite the growing body of literature examining acute PAP/PAPE responses, most studies have focused on immediate performance effects assessed shortly after a single conditioning activity, often under controlled or laboratory-based conditions [5,6,13]. Consequently, limited evidence exists regarding the chronic effects of repeatedly integrating strength-based exercises into routine warm-up structures over extended training periods, particularly in youth soccer populations [14,15]. Given that warm-ups are performed systematically throughout the training season, such an approach may represent a practical opportunity to induce meaningful long-term neuromuscular adaptations if appropriately designed.

Among these strategies, squat exercises are particularly relevant, as they target the main muscles involved in jumping and sprinting tasks [16,17]. The squat is a fundamental multi-joint movement for developing lower-limb power and explosive strength, qualities directly associated with soccer-specific actions, including short-distance sprints, vertical and horizontal jumps, and COD-based movements [18,19]. Although resistance training and plyometric programs have been extensively studied in soccer [20], the repeated integration of strength-oriented exercises into the warm-up as a long-term training stimulus remains underexplored, especially in youth players.

Previous studies indicate that incorporating heavy or explosive squats can enhance sprint and jump performance in trained athletes [18,21]. However, most existing research has examined these adaptations within dedicated strength sessions rather than embedded within the warm-up structure of routine soccer training [11,18,19,20]. Moreover, many studies have been conducted under controlled experimental conditions that may not fully reflect the organizational and ecological constraints of collective soccer warm-ups [22,23]. In youth soccer players, evidence remains limited and inconsistent. Some studies suggest that squat-induced activation can improve speed and explosive strength [21,24], while others report no significant effects, potentially due to differences in load, player experience, or recovery intervals [13].

Therefore, it is essential to assess the practical effectiveness of integrating squat exercises into the final phase of warm-up compared to traditional technical drills, such as the widely used “rondo”. The aim of this study was to examine the long-term effects of including squat exercises in the final phase of the warm-up on sprinting, jumping, COD, and aerobic performance in young soccer players. Aerobic performance was included to verify whether the warm-up interventions induced any unintended or differential adaptations beyond neuromuscular qualities and to confirm the specificity of potential training-induced effects, as strength-oriented interventions are generally not expected to meaningfully alter aerobic capacity [25]. We hypothesized that a squat-based warm-up applied twice weekly over nine weeks would induce greater chronic improvements in sprint, jump, and COD performance than a rondo-based warm-up, while aerobic performance would remain largely unchanged between groups.

## 2. Material and Methods

### 2.1. Participants

Before recruitment, an a priori sample-size estimation was conducted using G*Power (version 3.1.9.7; University of Kiel, Kiel, Germany) for a repeated-measures ANOVA (within–between interaction; group × time) [26,27]. With α = 0.05, power (1 − β) = 0.80, and an expected medium-to-large interaction effect (f = 0.35), the minimum required sample was 20 participants (10 per group). To allow for potential attrition, 24 male youth soccer players (under-17 category [U17]) from the same team were recruited, and all completed the study (0 dropouts). Players were stratified by playing position (central defender, full-back, midfielder, and forward) to ensure comparable representation of roles between groups. Within each position stratum, participants were then randomly allocated to the experimental group (EG; *n* = 12; age = 17.1 ± 0.6 years, height = 174.3 ± 5.8 cm, body mass = 66.5 ± 6.7 kg, soccer experience = 6.2 ± 1.1 years) or the control group (CG; *n* = 12; age = 17.0 ± 0.7 years, height = 173.6 ± 6.1 cm, body mass = 65.8 ± 7.1 kg, soccer experience = 6.0 ± 1.3 years) using a coin toss performed by an investigator not involved in testing. This ensured that randomization occurred within positions rather than across the full sample, thereby preventing positional imbalance between groups. Outcome assessments were performed by an assessor blinded to group allocation. Randomization and group assignment were managed by an investigator not involved in testing, and the assessor had no access to the allocation list; participants were identified using coded IDs during all testing sessions. For their regular training, players participated in five training sessions and one match per week.

Consistently with a random assignment, no significant differences between groups were found at baseline for age, height, body mass, and training experience (*p* > 0.05; independent *t*-test). The inclusion criteria were as follows: (i) all participants belonged to the same team; (ii) no player reported illness or injury during the eight weeks prior to the study or throughout the experimental period; (iii) all participants had prior experience performing the squat exercise; (iv) no physical or cognitive disorders were reported; and (v) participants attended training sessions regularly and consistently.

All participants and their legal guardians provided written informed consent, and the study was conducted in accordance with the Declaration of Helsinki and approved by the institutional ethics committee of the High Institute of Sports and Physical Education in Kef (ISSEPK-0036/2024, 1 December 2024). Table 1 presents baseline characteristics of the participants.

### 2.2. Experimental Design

The study was conducted over an eleven-week period, including a pre-test week, nine weeks of training, and a post-test week. Prior to the intervention, participants’ anthropometric characteristics and the one-repetition maximum (1-RM) were assessed. The 1-RM was estimated using a submaximal squat test, in which participants performed progressively heavier loads until reaching a weight they could lift for 4–6 repetitions with correct technique, then calculated using the Brzycki formula. This provided a safe and reliable basis for progressive load prescription. During the intervention period, the EG added targeted squat exercises at the end of the warm-up twice weekly to induce a post-activation potentiation effect and enhance neuromuscular readiness [10,22]. The CG, under the same time conditions, performed a 4v2 rondo drill [28] focused on ball retention and reactivity, without performing any resistance training exercise. Both groups then completed the same regular soccer training program, ensuring equal overall training volume and load. This design isolated the specific contribution of each warm-up modality to performance adaptations.

Performance assessments were conducted before and after the intervention using validated tests for youth soccer players: 10 m and 30 m sprints, standing long jump (SLJ), five-jump test (5JT), squat jump (SJ), countermovement jump (CMJ), T-half agility test, and the VAMEVAL test. Each participant completed two maximal trials per test (10 m, 30 m, SLJ, 5JT, SJ, CMJ) with three minutes of passive recovery, and the best performance was recorded. Testing sessions were standardized, with consistent verbal encouragement, and all measurements were taken on the same synthetic pitch between 16:30 and 17:00 to control for circadian influences [29]. A familiarization session was conducted beforehand to minimize learning effects. Rating of perceived exertion (RPE) was collected after each intervention to assess internal intensity [30].

### 2.3. Training Protocol

The nine-week training program consisted of five weekly sessions during the competitive season, with each session beginning with a standardized warm-up divided into three phases: an 8 min general phase including light jogging, joint mobility exercises, and dynamic stretching; an 8 min specific phase with coordination and acceleration drills using the ball; and a 10–12 min final phase consisting of intervention-specific exercises, either squat-based (EG) or rondo-based (CG) (Table 2).

The experimental intervention was performed twice weekly during the final phase of the warm-up. For the EG, this phase consisted of a structured squat protocol implemented during training sessions and not immediately before competitive matches, ensuring that it did not interfere with match performance. The warm-up used a progressive loading strategy that was carefully supervised and adapted for youth players with prior squat experience, allowing safe execution while promoting chronic muscular and motor adaptations. Intensity progressed during the course of the nine weeks from 50% to 85% of 1-RM following the principle of progressive overload. Each session included 3–4 sets of 4–12 repetitions performed at a 2-0-1 tempo (2 s eccentric, no pause, 1 s concentric explosively), with 2–3 min of rest between sets [31], resulting in a total duration of approximately 10–12 min for the final phase. In this exercise, the players started from an upright standing position with full extension of the hips and knees, while the barbell was positioned across the upper back at the level of the acromion. They then performed the downward phase until the thighs went below a 90° angle, followed by the upward phase to return to the starting position [32]. To ensure efficient organization and avoid time loss, four squat stations were installed along the sideline of the football pitch, each equipped with a barbell and rack positioned at shoulder height and spaced two meters apart. Four players performed the exercise simultaneously, followed by subsequent groups, until all participants completed their assigned sets. Recovery times were synchronized across players to standardize training conditions.

The CG performed a 4v2 rondo drill emphasizing ball retention and rapid short movements [28]. The rondo drill was performed in two bouts separated by 1 min of rest. The bout durations were progressively increased across the program: weeks 1–5: 1st bout = 4 min, 2nd bout = 5 min; weeks 6–9: 1st bout = 5 min, 2nd bout = 6 min, resulting in a total phase duration of 10–12 min, including the rest period. Both groups continued their regular football training to ensure an equivalent overall training volume (Table 3). Participants were instructed to maintain regular sleep patterns and avoid strenuous physical activity the day before testing to minimize fatigue.

### 2.4. Procedures

#### 2.4.1. Rating of Perceived Exertion

The internal training load was assessed immediately after the squat exercises (EG) or rondo drills (CG) performed during the final phase of the warm-up. The Borg CR-10 RPE scale was used to measure the perceived effort associated with each session [30]. This method has been previously shown to be valid and reliable for quantifying internal load in soccer contexts [33]. To ensure accurate responses, all participants were familiarized with the RPE scale prior to the intervention.

#### 2.4.2. Linear Sprint

Sprint performance was assessed over 10 m and 30 m on a synthetic grass pitch. Each participant performed two maximal 30 m sprints, with split times recorded at 10 and 30 m [34]. To guarantee uniformity and minimize any order effects, all tests were administered to each participant in the same standardized order before and after the intervention. A passive recovery period of three minutes was provided between each sprint to minimize fatigue. Sprint times were recorded using electronic timing gates (Globus, Microgate, Bolzano, Italy). For data analysis, the fastest time from the two trials was retained for both the 10 m split and the 30 m sprint. The sprint tests showed excellent reliability (10 m: ICC = 0.92, CV = 2.1%; 30 m: ICC = 0.93, CV = 1.9%).

#### 2.4.3. Horizontal Jump

Horizontal jump performance was assessed using the standing long jump and the five-jump test. In the SLJ, participants jumped forward as far as possible from a standing position with feet together, landing with both feet. In the 5JT, participants performed five consecutive horizontal jumps, emphasizing maximal distance and proper landing mechanics. Two trials were conducted for each test with three minutes of passive recovery, and the best performance was recorded. Reliability was good for both tests (SLJ: ICC = 0.88, CV = 3.0%; 5JT: ICC = 0.89, CV = 2.7%).

#### 2.4.4. Vertical Jump

Vertical jump performance was evaluated using the squat jump and countermovement jump [35]. Prior to the assessment, participants executed 2-3 self-paced submaximal CMJs and SJs to familiarize themselves with the testing procedures and to ensure adequate specific warm-up. During all trials, players were instructed to place their hands on their hips to eliminate the contribution of arm swing and minimize coordination effects, thereby isolating lower-limb extensor performance [35].

For the SJ, participants adopted a static semi-squat position with knees flexed at approximately 90° and performed a vertical jump without any preparatory movement. For the CMJ, they completed a rapid downward movement immediately followed by a forceful upward jump. Each participant performed two maximal attempts for both SJ and CMJ, with approximately two minutes of passive recovery between trials. The best performance was retained for analysis. Both tests showed high reliability (SJ: ICC = 0.90, CV = 2.5%; CMJ: ICC = 0.91, CV = 2.3%).

#### 2.4.5. Change-of-Direction

The COD performance was assessed using the T-half agility test [36]. Participants sprinted, shuffled, and backpedaled along a T-shaped course as quickly as possible. Two trials were performed with three minutes of passive recovery, and the best trial was retained for analysis. The test demonstrated good reliability (ICC = 0.87; CV = 2.8%).

#### 2.4.6. Aerobic Endurance

Aerobic endurance was assessed using the VAMEVAL test, which provides a reliable measure of maximal aerobic speed (MAS) [37]. The test was performed on a 200 m running track. The course was marked with ten cones placed at 20 m intervals, and participants followed a pre-programmed auditory signal (beep) to guide their running pace. The test began at 8 km·h^−1^ and increased by 0.5 km·h^−1^ every minute until volitional exhaustion. The test was terminated when a participant failed to reach the next cone in time with the beep on two consecutive occasions or stopped due to fatigue [37]. The highest speed successfully maintained before exhaustion was recorded as the MAS. Testing was conducted under stable environmental conditions (≈17–20 °C; 70–75% relative humidity; no wind) and at the same time of day for all sessions, making environmental influences on performance unlikely.

### 2.5. Statistical Analysis

All data were analyzed using SPSS version 28.0 (IBM, Armonk, NY, USA). Descriptive statistics are presented as mean ± standard deviation (mean ± SD). Normality of distribution was verified using the Shapiro–Wilk test, and homogeneity of variance was checked with Levene’s test. To examine the effects of the intervention, a mixed-design ANOVA (Group × Time) was performed for each physical performance variable, with time (pre- vs. post-intervention) as the within-subject factor and group (EG vs. CG) as the between-subject factor. When significant main effects or interactions were detected, Bonferroni post hoc tests were applied to identify pairwise differences between pre- and post-test values within and between groups.

Effect sizes were calculated using partial eta squared (η^2^p) for the ANOVA and interpreted according to Cohen’s guidelines as small (0.01), medium (0.06), or large (0.14) [38]. Within-group changes were quantified using percentage change (Δ%) and Cohen’s d, interpreted as trivial (<0.2), small (0.2–0.5), moderate (0.5–0.8), or large (>0.8) [38].

Session-RPE between-group differences (EG vs. CG) were tested using Mann–Whitney U tests. Because these comparisons were repeated across 18 training sessions, *p*-values were adjusted across sessions using the Holm–Bonferroni procedure to control the family-wise type I error rate (α = 0.05). Statistical significance was set at *p* < 0.05.

## 3. Results

**Figure 1:**
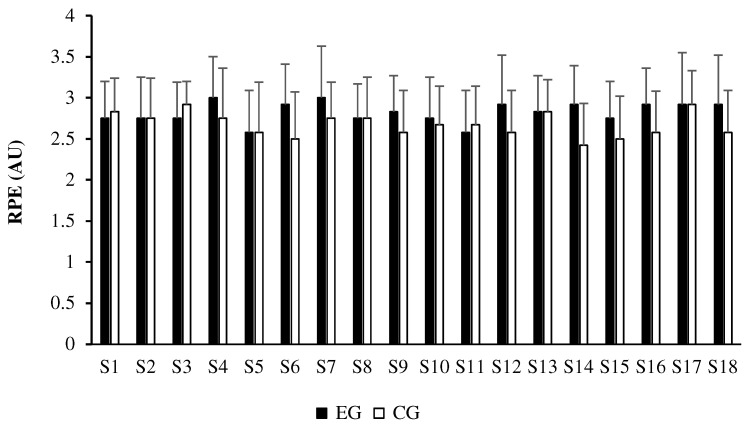
Post–warm-up session-RPE across the 18 training sessions in the experimental and control groups.

Mann-Whitney U tests showed that RPE values were generally similar between the experimental and control groups throughout the intervention, with no significant between-group differences observed in any session (all *p* ≥ 0.05; Table 4). Figure 1 illustrates the weekly RPE evolution, showing parallel trends between groups.

Pre- and post-intervention comparisons revealed significant improvements in EG across most physical performance tests, whereas CG showed no significant changes. Significant differences were observed for the EG in the 10 m sprint (*p* < 0.001), 30 m sprint (*p* < 0.001), SJ (*p* < 0.001), CMJ (*p* < 0.001), SLJ (*p* = 0.003), 5JT (*p* < 0.001), and T-half agility (*p* < 0.001) tests, indicating notable gains in acceleration/speed, jump, and COD performance.

In contrast, no significant changes were found for MAS performance (*p* > 0.05), suggesting no meaningful change in aerobic endurance. The CG did not show significant improvements for any variable (all *p* > 0.05). Mean values, percentage changes, and effect sizes are detailed in Table 5.

The results presented in Table 6 show that the two-way ANOVA revealed significant main effects of time for most physical performance variables (all *p* < 0.05), except for the MAS (*p* > 0.05). These findings indicate an overall improvement in performance over time, particularly in sprint, jump, and COD tests. No significant main effect of group was observed, suggesting that participants in the two groups exhibited comparable performance levels.

Conversely, significant Group × Time interaction effects were observed for 10 m and 30 m sprint, SJ, CMJ, SLJ, 5JT, and COD performance (all *p* < 0.05), indicating that the magnitude of improvement from pre- to post-test differed between groups. In contrast, no Group × Time interaction was found for the aerobic endurance performance (*p* > 0.05).

## 4. Discussion

The present study investigated the effects of integrating squat exercises into the final phase of warm-up on sprint, jump, COD, and aerobic endurance performance in youth soccer players over a nine-week intervention. The main findings indicate that EG showed significant improvements in short sprints (10 and 30 m), vertical and horizontal jumps, and COD, with moderate to large effect sizes. In contrast, no significant change was observed on aerobic endurance.

The EG improved significantly in the 10 m and 30 m sprint tests. These results suggest that incorporating squat exercises during warm-up can enhance the ability to generate and apply force rapidly over short distances, consistent with the expected effects of repeated resistance training exposure. In practical terms, the squat-integrated warm-up likely contributed to long-term adaptations in lower-limb strength and rapid force production through improvements in neuromuscular function [32,39,40,41,42]. Similar findings have been reported in the literature, where lower-limb resistance training improved sprint performance in soccer players [43]. The large effect observed in the 10 m sprint highlights the relevance of this stimulus for acceleration capacity, which is critical for match actions such as pressing, defensive recovery, and attacking runs [44,45].

Significant gains were also observed in jump performance, with SJ and CMJ performance showing the greatest improvements. These results indicate that regular exposure to squat-based resistance work may enhance lower-limb power and movement efficiency, likely through improved motor-unit recruitment and intermuscular coordination, thereby improving explosive performance [46]. Horizontal jump performance also improved, as reflected in SLJ and 5JT. The transfer of squat-based adaptations to both vertical and horizontal force production further supports the role of improved neuromuscular coordination and intermuscular efficiency [47]. Although some studies report that explosive strength exercises can acutely enhance sprint and jump performance when incorporated into warm-up routines [48], in the present study the observed pre-post improvements are better interpreted as the result of training-specific adaptations accumulated across nine weeks.

The T-half agility test performance improved significantly in the EG. COD performance depends on acceleration and deceleration capacities, which are strongly influenced by lower-limb strength/power and the ability to rapidly produce and absorb force [49,50,51]. Therefore, the repeated squat stimulus incorporated into the warm-up likely contributed to enhancing these qualities in a soccer-specific context [40,52].

No significant improvements were observed in aerobic endurance, indicating that squat-based warm-up primarily targets neuromuscular and explosive capacities rather than aerobic fitness. This limited impact on aerobic endurance performance can be explained by the nature, duration, and intensity of the squat intervention, which is unlikely to provide sufficient cardiovascular stimulus to elicit aerobic adaptations [53]. In general, improvements in aerobic endurance require longer-duration continuous or interval training with higher cardiovascular demand [54,55,56]. Overall, these results align with the principle of training specificity: resistance-based interventions preferentially improve sprint-, jump-, and COD-related qualities, with limited transfer to endurance performance [25].

### 4.1. Practical Applications

Practically, integrating a low-volume squat component into the warm-up can be used as a training-session strategy to progressively develop sprint-, jump-, and COD-related qualities across a season. However, the loads used in the present intervention (up to 85% 1RM) should not be interpreted as a recommendation for a routine pre-match warm-up in youth players. When applied, strength-based warm-ups should be implemented only in athletes familiar with resistance training and under qualified supervision, with careful attention to technical execution and fatigue management. If coaches choose to use a competition-day activation approach, it should be individualized, conservative (lower volume and/or lower loads), and trialed during training first, given the mixed evidence on heavy potentiation protocols and their limited added benefit beyond standard warm-up effects [57,58,59]. In line with recent youth-soccer evidence showing benefits of distributing neuromuscular training across multiple weekly exposures [12], coaches may implement warm-up–integrated strength/power work as brief, repeated exposures across the week (e.g., 2–3 sessions·week^−1^) rather than concentrating volume into a single session.

### 4.2. Limitations and Future Directions

Several limitations should be acknowledged. First, integrating loaded squats into the warm-up likely increased the total resistance-type stimulus in the experimental group compared with the control condition. Importantly, the control group performed rondo drills, which are primarily technical–tactical exercises and are not equivalent in strength-loading demands; therefore, between-group differences may reflect the overall nature/type of exercise stimulus and its loading characteristics, rather than the inclusion of squats alone. Second, biological maturation was not assessed (e.g., peak height velocity status), which may have influenced training responsiveness in this age group. Third, although performance outcomes improved, we did not include a direct post-intervention measure of strength (e.g., 1RM, isometric strength, or force–time characteristics), which limits mechanistic interpretation of the observed changes. Fourth, training-load monitoring was limited to post–warm-up session-RPE and did not capture overall weekly internal and external loads (including training and match demands), which could confound adaptation patterns across the intervention. Finally, participants were male U17 players from a single team; thus, generalization to female athletes, older age groups, and adult or professional players should be made with caution. Future research should examine whether combining squat-based warm-ups with other neuromuscular activation strategies (e.g., plyometrics or resisted sprints) produces greater performance benefits than any single modality. In addition, integrating technical drills with strength-oriented elements may improve transfer to game-specific scenarios [23,60,61].

## 5. Conclusions of the Discussion

In summary, integrating squat exercises into the final phase of warm-up over nine weeks led to moderate-to-large improvements in sprint, jump, and COD performance in youth soccer players, while no meaningful change was observed in aerobic endurance. These findings suggest that a squat-integrated warm-up can serve as a practical and time-efficient training strategy to enhance key neuromuscular qualities relevant to match performance. The observed benefits are best interpreted as chronic training adaptations to a progressive strength stimulus embedded within routine practice, supporting the use of evidence-based warm-up designs to optimize physical performance in young athletes.

## Figures and Tables

**Table 1:** Baseline characteristics of the participants (Mean ± SD).

Variable	EG (*n* = 12)	CG (*n* = 12)	*p*-Value
Age (years)	17.1 ± 0.6	17.0 ± 0.7	0.72
Height (cm)	174.3 ± 5.8	173.6 ± 6.1	0.81
Body mass (kg)	66.5 ± 6.7	65.8 ± 7.1	0.84
Training experience (years)	6.2 ± 1.1	6.0 ± 1.3	0.69

EG = Experimental Group, CG = Control Group, kg = kilograms, cm = centimeters, *n* = number of participants, Data are presented as mean ± standard deviation.

**Table 2:** Description of the intervention program.

Session Component	EG	CG
General warm-up (8 min)	Jogging, mobility, dynamic stretching	Jogging, mobility, dynamic stretching
Specific warm-up (8 min)	Technical drills with the ball	Technical drills with the ball
Final phase (10–12 min)	3–4 sets × 4–12 repetitions of squats with progressive load (50–85% 1-RM), tempo 2-0-1, 2 min rest between sets	2 bouts of 4v2 rondo drill, 1 min rest; bout durations progressively increased across 9 weeks: Weeks 1–5: 1st bout = 4 min, 2nd bout = 5 min Weeks 6–9: 1st bout = 5 min, 2nd bout = 6 min
Total session duration	26–28 min	26–28 min

EG = Experimental Group, CG = Control Group, min = minute.

**Table 3:** Structure and progression of the squat-integrated warm-up protocol.

Week	Day	%1-RM	Reps Per Set	Sets	Tempo (Eccentric/Concentric)	Notes
1	Tue, Thu	50%	12	3	2-0-1	Focus on technique, controlled depth
2	Tue, Thu	55%	10	3	2-0-1	Slightly higher intensity
3	Tue, Thu	60%	10	3	2-0-1	Maintain good form, moderate load
4	Tue, Thu	65%	8	3	2-0-1	Emphasize power on concentric phase
5	Tue, Thu	70%	8	3	2-0-1	Full explosive concentric phase
6	Tue, Thu	75%	6	3	2-0-1	Focus on maximal force output
7	Tue, Thu	75%	6	4	2-0-1	Increase volume with extra set
8	Tue, Thu	80%	5	4	2-0-1	High-intensity phase, low reps, explosive
9	Tue, Thu	85%	4	4	2-0-1	Peak phase, emphasis on maximal power

Week = week number, Day = training day, %1-RM = percentage of one-repetition maximum, Reps per Set = repetitions per set, Sets = number of sets, Tue = Tuesday, Thu = Thursday. Tempo notation: Eccentric (down)-Pause-Concentric (up) → e.g., 2-0-1 = 2 s down, 0 pause, 1 s up explosive.

**Table 4:** Mann–Whitney U statistics and *p*-values for between-group (EG vs. CG) comparisons of session-RPE across the 18 training sessions.

Session	U	*p*-Value
S1	66	0.66
S2	60	0.39
S3	54	0.18
S4	50.5	0.13
S5	66	0.75
S6	54	0.27
S7	58	0.34
S8	60	0.39
S9	63	0.56
S10	66	0.68
S11	54	0.18
S12	60	0.41
S13	66	0.66
S14	66	0.68
S15	60	0.42
S16	66	0.68
S17	50	0.13
S18	62	0.48

**Table 5:** Pre- and post-intervention values (mean ± SD) for all outcomes in the experimental and control groups.

Variable	Group	Pre-Test	Post-Test	Δ%	Cohen’s d	Effect
Sprint 10 m (s)	EG	1.81 ± 0.04	1.77 ± 0.05	−2.21	1.0	Large
	CG	1.78 ± 0.06	1.79 ± 0.06	0.56	0.16	Trivial
Sprint 30 m (s)	EG	4.37 ± 0.12	4.30 ± 0.11	−1.60	0.58	Medium
	CG	4.33 ± 0.11	4.32 ± 0.09	−0.23	0.09	Trivial
SJ (cm)	EG	32.75 ± 2.63	36 ± 2.13	9.92	1.23	Large
	CG	34 ± 3.13	33.9 ± 2.19	−0.29	0.03	Trivial
CMJ (cm)	EG	35.6 ± 2.34	39.9 ± 1.72	12.08	1.83	Large
	CG	36 ± 2.33	36.08 ± 1.83	0.22	0.03	Trivial
SLJ (m)	EG	2.24 ± 0.2	2.4 ± 0.13	7.14	0.8	Large
	CG	2.25 ± 1.88	2.26 ± 0.12	0.44	0.05	Trivial
5JT (m)	EG	11.63 ± 0.83	11.9 ± 0.84	2.33	0.32	Small
	CG	11.99 ± 0.94	12.04 ± 0.86	0.42	0.05	Trivial
COD (s)	EG	5.66 ± 0.25	5.58 ± 0.24	−1.41	0.32	Small
	CG	5.77 ± 0.25	5.76 ± 0.24	−0.17	0.04	Trivial
MAS (km·h^−1^)	EG	17.25 ± 0.91	17.41 ± 0.84	0.93	0.18	Trivial
	CG	17.29 ± 0.72	17.33 ± 0.74	0.23	0.06	Trivial

Δ% = percentage change from pre- to post-test, Cohen’s d interpreted as. trivial (0–0.20), small (>0.20–0.50), Medium (>0.50–0.80), and large (>0.80). EG = Experimental Group; CG = Control Group; SJ = Squat Jump; CMJ = Countermovement Jump; SLJ = Standing Long Jump; 5JT = 5-Jump Test; COD: change-of-direction; MAS: maximal aerobic speed. m = meters, (km·h^−1^) = kilometers per hour, s = seconds, cm = centimeters.

**Table 6:** Mixed repeated-measures ANOVA results (group, time, and group × time interaction) for all outcomes.

Variable	Main Effect of the Time		Main Effect of the Group		Interaction Effect	
	F	η^2^p	F	η^2^p	F	η^2^p
**10 ** **m sprint (s)**	17.17 ***	0.48	0.43	0.02	7.1 *	0.24
**30 ** **m sprint (s)**	28.15 ***	0.56	0.11	0.05	14.61 **	0.39
**Squat jump (cm)**	45.38 ***	0.67	0.17	0.08	50.28 ***	0.69
**CMJ (cm)**	84.98 ***	0.79	4.59	0.17	78.57 ***	0.78
**SLJ (cm)**	11 ***	0.33	1.66	0.07	7.36 *	0.25
**5JT (m)**	35.14 ***	0.61	0.49	0.02	16.45 ***	0.42
**COD (s)**	42.01 ***	0.65	2.67	0.19	28.95 ***	0.56
**MAS (km·h^−1^)**	1.47	0.06	0.004	0.000	0.53	0.02

CMJ = Countermovement Jump; SLJ = Standing Long Jump; 5JT = 5-Jump Test; COD: change-of-direction; MAS: maximal aerobic speed. *: *p* < 0.05; **: *p* < 0.01; ***: *p* < 0.001.

## Data Availability

The data that support the findings of this study are openly available upon request from the corresponding author.
